# P02-11 Effectiveness and implementation of a long-term home-based exercise training programme using minimal equipment in COPD patients: A multi-centre randomised controlled trial

**DOI:** 10.1093/eurpub/ckac095.030

**Published:** 2022-08-29

**Authors:** Anja Frei, Thomas Radtke, Kaba Dalla Lana, Patrick Brun, Thomas Sigrist, Marc Spielmanns, Thomas Riegler, Gilbert Büsching, Tamara Cerini, Milo Puhan

**Affiliations:** 1 Epidemiology, Biostatistics and Prevention Institute, University of Zurich, Zurich, Switzerland; 2 Berner Reha Zentrum, Heiligenschwendi, Switzerland; 3 Departement for Pulmonary Medicine, Clinic Barmelweid, Barmelweid, Switzerland; 4 Pulmonary Medicine, Zürcher RehaZentren Wald, Wald, Switzerland

**Keywords:** COPD, home-based exercise training, randomised controlled trial, effectiveness, implementation evaluation

## Abstract

**Background:**

Although exercise training is an important component of pulmonary rehabilitation (PR) in chronic obstructive pulmonary disease (COPD), a majority of COPD patients fails to maintain training after PR. This study aimed to evaluate the effectiveness and implementation of a 12-months home-based, minimal equipment strength exercise programme.

**Methods:**

Parallel arm, multicentre study across four Swiss PR clinics, random allocation of COPD patients (1:1 ratio) into intervention (IG) or control group (CG, usual care). Primary outcome was change in dyspnoea (Chronic Respiratory Questionnaire, CRQ) from baseline to 12-months, secondary outcomes change in exercise capacity (1-minute-sit-to-stand-test [1-min-STST], 6-minute-walk-test [6MWT]), health-related quality of life, exacerbations and symptoms. Main effectiveness analyses were based on the intention-to-treat approach and adjusted linear regression models were used. To assess the implementation outcomes dose, reach, fidelity, adherence, acceptability and appropriateness, we conducted interviews with patients, coaches and stakeholder and analysed reports, diaries and notes.

**Results:**

123 patients (IG: 61, CG: 62) were randomised, 61 females, mean (*SD*) age 66.8 (8.1) years, and 104 participants completed 12-months follow-up (IG: 53, CG: 51). Of 53 IG participants, 37 (70%) conducted the training until study end. We found no difference in change of CRQ dyspnoea over 12 months (adjusted mean difference 0.28, 95% CI -0.23-0.80, p = 0.27). We found moderate evidence for a difference in 1-min-STST repetitions favouring the IG (adjusted mean difference 2.6 (95% CI 0.22-5.03, p = 0.033) but no evidence for an effect in other outcomes.

All involved groups perceived the strength-training exercises as appropriate, efficient for COPD patients and relevant to maintain improvements after PR. The patients' most important facilitators for long-term motivation were self-perceived improvement in strength, supervision by a coach and integration of the training in daily routine. Based on these insights, we redesigned and reworded the training material and introduced three new exercises.

**Conclusions:**

The exercise program had no effect on dyspnoea but improved 1-min-STST performance and patient-perceived fitness. The results from the insights of the involved persons enabled us to optimize the program for sustainable further use in clinical and other settings and inform the future design of patient-centred home-based exercise programs in COPD.

